# Reference Group Choice and Antibiotic Resistance Outcomes

**DOI:** 10.3201/eid1006.020665

**Published:** 2004-06

**Authors:** Keith S. Kaye, John J. Engemann, Essy Mozaffari, Yehuda Carmeli

**Affiliations:** * Duke University Medical Center, Durham, North Carolina, USA;; † Pfizer, New York, New York, USA;; ‡ Beth Israel Deaconess Medical Center, Harvard Medical School, Boston, Massachusetts, USA

**Keywords:** outcomes, antimicrobial resistance, methodology, Staphylococcus aureus, Enterococcus, cost, virulence, vancomycin, methicillin

## Abstract

Two types of cohort studies examining patients infected with methicillin-resistant *Staphylococcus aureus* (MRSA) and vancomycin-resistant enterococci (VRE) were contrasted, using different reference groups. Cases were compared to uninfected patients and patients infected with the corresponding, susceptible organism. VRE and MRSA were associated with adverse outcomes. The effect was greater when uninfected control patients were used.

Although several investigators have performed outcomes studies of patients infected or colonized with antimicrobial resistant bacteria, the design and interpretation of results with various methods has not been discussed (*1*). Typically, these outcomes studies use a cohort design and study patients infected with resistant bacteria (the exposure of interest for cases), who are compared either to patients without infection selected from a similar population (*2**–**6*) or to patients infected with corresponding, susceptible bacteria (e.g., comparing patients with methicillin-resistant *Staphylococcus aureus* [MRSA] to patients with methicillin-susceptible *S. aureus* [MSSA]) (*7**–**13*) (Appendix). When cases are compared to an uninfected reference group or "control group," the effect of a new, antimicrobial-resistant bacterial infection is assessed. When case-patients are compared to reference patients or "controls" infected with the corresponding susceptible bacteria, the impact of acquiring a resistance determinant is measured. Both types of comparison are valid and important, but they address different clinical scenarios.

We examined how the choice of the reference group might influence results of outcomes studies pertaining to antimicrobial resistant bacteria. We compared and contrasted the results of outcomes cohort studies for resistant bacteria by using the two different reference groups discussed previously. We used results from original studies of MRSA and vancomycin-resistant enterococci (VRE) (*9**,**10*) that initially used one reference group. In our study, we performed additional analyses comparing case-patients to different reference patients and contrasted the results.

## The Study

Both MRSA and VRE studies were designed as cohort studies and are discussed in detail elsewhere (*9**,**10*) (Appendix). Cases were defined as patients with MRSA surgical site infection (SSI) (i.e., the exposure of interest for study 1) and VRE wound infection (i.e., the exposure of interest for study 2). In each study, two different reference groups were used in separate analyses. Control group A included patients who did not have an infection caused by the target pathogen (*S. aureus* or enterococci). Control group B included patients with infection caused by the susceptible phenotype of the target pathogen (i.e., MSSA and vancomycin-susceptible enterococci [VSE]).

In both studies, three outcomes were examined: death, length of hospital stay, and total hospital charges. Hospital charges were variable direct charges obtained from hospital financial databases and are a surrogate for cost. Hospital costs were estimated using a cost-to-charge ratio of 0.7 (*14*).

Outcomes studies of antimicrobial drug resistance are notoriously hard to perform because of confounding variables related to underlying coexisting conditions (*1*). To control for confounding, we analyzed several variables, including individual coexisting conditions, the Charlson score, the American Society of Anesthesiologists-Physical Status (ASA) score, and duration of hospitalization before infection (Appendix). These variables were analyzed in multivariable analysis. Each of the outcomes was analyzed independently. The inverse log value was calculated for β coefficients of variables included in the predictor models, and these effect measures were described as the odds ratio (OR) for death rate and the multiplicative effects (ME) on length of stay and charges.

In the analysis comparing patients with SSI caused by MRSA to uninfected controls, the study cohort included 314 patients: 121 MRSA SSI cases and 193 uninfected surgical controls (Appendix). In multivariable analysis, MRSA SSI was significantly associated with death (OR = 11.4, p < 0.001). In the analysis comparing patients with MRSA SSI to patients with SSI caused by MSSA, the same 121 MRSA case-patients were compared to 165 control-patients with MSSA SSI. In multivariable analysis, MRSA SSI was significantly associated with death (OR = 3.4, p = 0.003). Additional covariates included in the adjusted models for death are listed in the footnotes of Table 1 and are discussed in the Appendix. The effect of MRSA on deaths was approximately threefold greater for the analysis using uninfected controls than for the analysis using MSSA controls.

**Table 1:** Outcomes and adjusted analyses for MRSA for study 1^a^

Outcome	Cases	Controls	Adjusted analyses		
			OR (95% CI)^b^	Attributable to MRSA	p value
Three analyses comparing MRSA cases (n = 121) and uninfected controls (n = 193)					
Deaths	20.7%	2.1%	11.4 (2.8 to 34.9)^c^	–	<0.001
Hospital days after surgery, mean per case	29.1	6.1	3.2 (2.7 to 3.7)^d^	13.4	<0.001
Charges ($), mean/case	118,414	34,395	2.2 (2.0 to 2.6)^e^	41,274	<0.001
Three analyses comparing MRSA cases (n = 121) and MSSA controls (n = 165)					
Deaths	20.7%	6.7%	3.4^f^	–	0.003
Hospital days after infection, mean per case	22.0	13.2	1.2^g^	2.6	0.11
Charges ($), mean per case	118,414	73,165	1.2^h^	13,901	0.03

^a^OR, odds ratio; CI, confidence interval; MRSA, methicillin-resistant *Staphylococcus aureus*; MSSA, methicillin-susceptible *S. aureus*. ^b^Odds ratio for deaths, and multiplicative effect (ME) for continuous outcomes (length of stay and charges). ^c^Adjusted for American Society of Anesthesiologists-Physical Status (ASA) score >3 and age. ^d^Adjusted for ASA score, duration of surgery, hospital, renal disease, diabetes, and length of hospital stay before surgery. ^e^Adjusted for ASA score, hospital, duration of surgery, renal disease, length of hospital stay, and intensive care unit (ICU) stay before surgery. ^f^Adjusted for ASA score >3, duration of surgery, and age. ^g^Adjusted for ASA score, renal disease, diabetes mellitus, hospital, duration of surgery, and length of stay before infection. ^h^Adjusted for ASA score, duration of surgery, length of hospital and ICU stay before infection, hospital, renal disease, and diabetes.

In the analysis comparing patients with SSI caused by MRSA to uninfected controls, multivariable modeling demonstrated that MRSA SSI was significantly associated with an increased length of stay (ME = 3.2, p < 0.001). Having an MRSA SSI was associated with an average adjusted attributable increase of 13.4 hospital days per case. In the analysis comparing patients with MRSA SSI to controls with SSI due to MSSA, a trend was seen toward an association between MRSA SSI and total hospital days (ME = 1.20, p = 0.11). Methicillin resistance was associated with an average adjusted attributable increase of 2.6 days per case, although this did not reach statistical significance. Additional covariates included in the adjusted models for length of stay are listed in the footnotes of Table 1 and are discussed in the Appendix. The effect of MRSA on length of stay was approximately threefold greater (11 days) for the analysis using uninfected controls than for analysis B using MSSA controls.

In the analysis comparing patients with SSI due to MRSA to uninfected controls, multivariable modeling showed that MRSA SSI was significantly associated with increased hospital charges (ME = 2.2, p < 0.001). MRSA was associated with mean adjusted additional attributable charges of $41,274 per case and an attributable cost of $28,891 per case. In the analysis comparing patients with SSI due to MRSA to controls with SSI due to MSSA, MRSA was significantly associated with increased hospital charges (ME = 1.2, p = 0.03). Methicillin resistance was associated with mean adjusted additional attributable charges of $13,901 per MRSA SSI case and an attributable cost of $9,731 per case. Additional covariates included in the adjusted models for cost are listed in the footnotes of Table 1 and are discussed in the Appendix. The effect of MRSA on cost was approximately twofold greater ($15,000) for the analysis using uninfected controls than for the analysis using controls with SSI due to MSSA.

In the analysis comparing patients with wound infection due to VRE to uninfected controls, 99 patients with VRE wound infection were compared to 280 matched controls who were not infected with enterococci (Appendix). In adjusted analysis, VRE wound infection was not an independent predictor of deaths (OR 2.0, p = 0.13). In the analysis comparing patients with wound infection due to VRE to control patients with wound infection due to VSE, the same 99 VRE wound infection cases were compared to 213 control patients with VSE wound infections. In multivariable analysis, VRE was significantly associated with mortality (OR 2.5, p = 0.04). Additional covariates included in the adjusted models for death rates are listed in the footnotes of Table 2 and are discussed in the Appendix. The magnitude of effect of VRE on deaths was similar for both analyses.

**Table 2:** Outcomes and adjusted analyses for vancomycin-resistant enterococci (VRE) in study 2

Outcome	Cases	Controls	Adjusted analyses		
			OR^a^	Attributable to VRE	p value
Three analyses comparing VRE patients (n = 99) and uninfected controls (n = 280)					
Deaths	12.1%	6.1%	2.0^b^	–	0.13
Length of stay (d), mean per case	15.2	8.5	1.8^c^	6.2	<0.001
Charges ($), mean per case	46,660	27,224	1.5^d^	13,884	<0.001
Three analyses comparing VRE patients (n = 99) and vancomycin-susceptible enterococci (VSE) controls (n = 213)					
Deaths	12.1%	6.6%	2.5^e^	–	0.04
Length of stay (d), mean per case	15.2	13.6	1.0^f^	–	0.5
Charges ($), mean per case	46,600	31,915	1.4^g^	12,766	<0.001

^a^Odds ratio for deaths, and multiplicative effect (ME) for continuous outcomes (length of stay and charges). ^b^Adjusted for number of comorbid illnesses and admission to the intensive care unit (ICU). ^c^Adjusted for propensity score (i.e., likelihood of being a VRE patient [Appendix]), being transferred from another institution, renal disease, malignancy, and admission to the ICU. ^d^Adjusted for propensity score (i.e., likelihood of being a VRE patient), having had surgery before cohort inclusion, and duration of hospitalization before cohort inclusion. ^e^Adjusted for surgery, sex, and admission to the ICU. ^f^Adjusted for duration of hospitalization before cohort inclusion, admission to the ICU, and malignancy. ^g^Adjusted for having had surgery before inclusion in the cohort, and duration of hospitalization before cohort inclusion.

In the analysis comparing patients with wound infection due to VRE to uninfected controls, multivariable modeling showed a significantly longer duration of hospitalization after inclusion in the cohort for VRE cases than for controls not infected with enterococci (ME 1.8, p < 0.001, average adjusted attributable increase of 6.2 days in length of stay). In the analysis comparing patients with wound infection due to VRE to control patients with VSE wound infection, length of stay after isolation of enterococci was similar among VRE cases and VSE controls (mean of 15.2 vs. 13.6 days, p = 0.5) and the differences in length of stay remained non-significant in multivariate analysis (ME = 1.0, p = 0.5). Additional covariates included in the adjusted models for length of stay are listed in the footnotes of Table 2 and are discussed in the Appendix. The effect of VRE on length of stay was approximately twofold greater (6 days) for the analysis using uninfected controls than for the analysis that used VSE controls.

In the analysis comparing patients with wound infection due to VRE to uninfected controls, multivariable modeling demonstrated that VRE cases generated significantly greater hospital charges than controls (adjusted ME = 1.5, p < 0.001, mean adjusted additional attributable charges of $13,884 per VRE wound infection and attributable cost of $9,719 per infection). In the analysis comparing patients with wound infection due to VRE to controls with VSE wound infection, VRE wound infection was associated with increased hospital charges (ME = 1.4, p < 0.001, average adjusted additional attributable charges of $12,766 per infection and attributable cost of $8,936 per infection). Additional covariates included in the adjusted models for cost are listed in the footnotes of Table 2 and are discussed in the Appendix. The effect of VRE on cost was similar in both analyses.

## Conclusions

We examined how the criteria used to select a reference group (i.e., a comparison or control group for cases) influenced outcomes study results. Two types of control patients were studied, and in both types of analyses, VRE and MRSA were associated with significant, adverse clinical outcomes. In general, the effects (i.e., OR or ME) were of greater magnitude when controls not infected with the target organism (and thus representative of a random sample of the source population) were used. This is logical since analyses using uninfected controls assess the effect of acquiring a new infection and a resistant pathogen. When patients who are infected with a susceptible organism are used as controls, the analysis quantifies only the effect of acquiring a resistance trait.

The differences in results between the two analyses were much greater for the MRSA SSI study than for the VRE wound infection study. The impact on clinical outcomes was two- to threefold greater when patients with MRSA SSI were compared to an uninfected control group as opposed to comparison with control patients infected with MSSA SSI. In contrast, when patients with VRE wound infection were compared to uninfected patients, similar results were obtained as when patients with VSE wound infections were used as controls. We believe that the magnitude of differences in results for the two analyses is directly related to the virulence of the infecting organism (Appendix).

The studies were performed in two different geographic locales and by using slightly different analytic methods. While this is a limitation in that cost results are not directly comparable, we feel including these two studies improves the generalizability of our results and strengthens our findings.

For studies of antimicrobial resistance, a reference group must be chosen on the basis of the investigators' objective. From a public health perspective, results from outcomes studies pertaining to antimicrobial resistance are frequently used to help allocate resources for interventions. If the objective of a study is to investigate the independent effects of a resistance trait or phenotype (e.g., methicillin resistance), then the most appropriate control group would consist of patients infected with a susceptible corresponding organism. If the goal is to assess the effect of a new infection caused by a particular pathogen, uninfected control patients would be the preferable comparison group. Alternatively, a complete analysis might include both types of control groups; this analysis would allow the reader to assess the effect of acquiring a resistance phenotype alone and the impact of acquiring a new infection caused by a resistant bacteria.

## Appendix

Several studies have analyzed attributable outcomes of patients infected with resistant bacteria who are compared to reference patients infected with corresponding, susceptible bacteria (15–46). We have conducted two studies, both at teaching hospitals. Study 1 (methicillin-resistant *Staphylococcus aureus* [MRSA]) was conducted at Duke University Medical Center, a 900-bed tertiary care academic medical center, and Durham Regional Hospital, a 350-bed community hospital, both located in Durham, North Carolina. Study 2 (vancomycin-resistant enterococci [VRE]) was conducted at Beth Israel Deaconess Medical Center, West Campus, a 320-bed urban tertiary care teaching hospital in Boston, Massachusetts. In both studies, data were abstracted from various sources, including computerized hospital databases (e.g., accounting, administrative, infection control, and microbiology databases) and patient medical records and were compiled into a single dataset (Access, Microsoft Corp., Redmond, WA). In both studies, organisms were identified from clinical specimens by using standard microbiologic methods that are in accordance with the National Committee for Clinical Laboratory Standards guidelines. Exact methods of data collection, assembly, and microbiology are described elsewhere (*47**-**48*).

To control for confounding, we used multivariable analysis, examining each of the outcomes independently. The following variables were analyzed as potential confounders: patient demographics, admitting diagnosis, coexisting conditions, and number of days in hospital and intensive care before cohort inclusion. For study 2, propensity score for likelihood of being a VRE case (*49*), having a major surgical procedure, and being infected with *Clostridium difficile* or MRSA were also analyzed.

Statistical analysis was performed on Stata (Stata Corp., College Station, TX) software and/or on SAS 8.1 (Cary, NC). Age was analyzed with the Student *t* test. and other continuous and ordinal variables were compared with the two-sided Wilcoxon rank sum test. The Fisher exact test was used to analyze dichotomous variables. Spearman correlation coefficients were calculated to detect trends among continuous variables (e.g., between length of hospital stay and continuous independent variables and between cost and continuous independent variables). Matched analyses were used in study 2, for the analysis comparing VRE wound infection to control patients with VSE wound infection (*47**,**50*).

Each outcome was examined independently, with multivariate analysis. In both studies, death rates were analyzed with logistic regression (conditional maximum-likelihood in the VRE study, to account for matching) and hospital charges with linear regression. For the MRSA study, total hospital days after infection were analyzed by using linear regression. For length of hospital stay, semiparametric survival models with accelerated failure time (Weibull) were used for the VRE analysis.

For multivariate linear regression, the following data transformation was performed. In the MRSA study, cost and length of hospital stay were log transformed and for the VRE study, cost was log transformed. No log transformation was performed for logistic regression and survival analyses, and no log transformation was performed for univariate or bivariate analyses.

The inverse log value was calculated for β coefficients of variables included in the predictor models, and these effect measures were described as multiplicative effects (ME) on length of stay and cost. All statistical tests were two-tailed. A p <0.05 was considered significant.

Adjusted mean attributable outcomes per resistant infection (VRE and MRSA) were calculated as follows for hospital days and charges. Charges per VRE infection are used as an example:

Mean attributable charges per VRE infection = [(mean charges for control patients) x (inverse log of β coefficient for adjusted VRE infection variable)] – (mean charges for control patients)

Three groups were studied: 121 MRSA surgical site infection (SSI) cases, 193 uninfected surgical controls, and 165 control patients with MSSA SSI. Descriptive characteristics of these groups and results of bivariate analyses are in Table A1.

In the analysis comparing patients with MRSA SSI to uninfected controls, in addition to MRSA, significant predictors of mortality included the American Society of Anesthesiologists-Physical Status (ASA)score >3 and age >73 (Table A2). When patients with MRSA SSI were compared to control patients with MSSA SSI, in addition to MRSA, significant predictors of death included ASA score >3 and age >61 years. This model was controlled for the confounding effects of operative duration (Table A3).

In the analysis comparing patients with MRSA SSI to uninfected controls, in addition to MRSA, other predictors of increased length of hospital stay included ASA score, duration of surgery, and length of hospital stay before surgery. This model was controlled for the confounding effects of admission to the tertiary care hospital, diabetes, and renal disease (Table A2). When patients with MRSA SSI were compared to control patients with MSSA SSI, significant predictors of increased length of hospital stay included ASA score, renal disease, duration of surgery, and length of stay before infection. This model was controlled for the confounding effects of diabetes mellitus and admission to a tertiary care hospital (Table A3).

In the analysis comparing patients with MRSA SSI to uninfected controls, in addition to MRSA, other predictors of increased cost included ASA score, admission to tertiary care hospital, duration of surgery, length of hospital stay, and intensive care unit (ICU) stay prior to surgery. This model was controlled for the confounding effect of renal disease (Table A2). When patients with MRSA SSI were compared to control patients with MSSA SSI, significant predictors of increased cost, in addition to MRSA, included ASA score, duration of surgery, length of hospital and ICU stay before infection, and admission to a tertiary care hospital. This model was controlled for the confounding effects of renal disease and diabetes (Table A3).

Three groups of patients were studied: 99 VRE case patients with wound infection, 280 matched controls who were not infected with enterococci, and 213 control patients with VSE wound infections. Descriptive characteristics and results of bivariate analyses are in Table A4.

In the analysis comparing patients with VRE wound infection to uninfected controls, the impact of VRE wound infection on deaths was controlled for the confounding effects of number of comorbid illnesses and admission to the intensive care unit (ICU) (Table A5). When patients with VRE wound infection were compared to control patients with VSE wound infection, significant predictors of deaths included admission to the ICU. This model was controlled for the confounding effects of surgery and sex (Table A6).

In the analysis comparing patients with VRE wound infection to uninfected controls, predictors of increased length of hospital stay, in addition to VRE, included being transferred from another institution, renal disease, malignancy, and admission to the ICU. This model was controlled for the confounding effect of propensity score (i.e., likelihood of having a case of VRE)] (Table A5). When patients with VRE wound infection were compared to control patients with VSE wound infection, significant predictors of increased length of stay included admission to the ICU. This model was controlled for the confounding effects of duration of hospitalization before cohort inclusion and malignancy (Table A6).

In the analysis comparing patients with VRE wound infection to uninfected controls, predictors of increased cost, in addition to VRE, included having had surgery before cohort inclusion (Table A5). This model was controlled for the confounding effects of propensity score (i.e., likelihood of being a VRE case) and duration of hospitalization before cohort inclusion. When patients with VRE wound infection were compared to control patients with VSE wound infection, significant predictors of increased cost, in addition to VRE, included having had surgery before inclusion in the cohort. This model was controlled for the confounding effect of time in hospital before cohort inclusion.

The differences in results between the two analyses are much greater for a virulent primary pathogen than for a nonvirulent, secondary invader. When a virulent pathogen is studied (e.g., *S. aureus*), the infected susceptible group (MSSA) is at much greater risk for adverse clinical outcomes than the uninfected control group, and analyses comparing resistant cases (MRSA) to these two control groups produce notably different results. Enterococci are often nonvirulent secondary invaders (e.g., colonizers) in wound infections and are frequently part of a mixed flora of infecting pathogens rather than true primary pathogens. The results obtained when patients with VRE wound infection were compared to patients not infected with enterococci were similar to results obtained when patients with VSE wound infections were used as controls. In our opinion, when resistant pathogens of low virulence (e.g., VRE in wounds) are analyzed, the infected susceptible (e.g., VSE) and uninfected control groups approximate one another, and results of analyses comparing resistant cases to these two control groups are similar.

## Appendix

**Table A1:** Study 1, patient characteristics, methicillin-resistant *Staphylococcus aureus* (MRSA), controls not infected with *S. aureus* and controls with methicillin-susceptible *S. aureus* (MSSA) surgical site infections, bivariable analyses

Variable	Cases, MRSA (%) (n = 121)	Controls, uninfected patients (%) (n = 193)	p value, (MRSA vs. uninfected controls)	Controls, MSSA (%) (n = 165)	p value (MRSA vs. MSSA)
Age, mean ± SD, y	63.9 ± 15.4	57.3 ± 18.3	0.001	55.1 ± 17.4	<0.001
Male sex	55 (45.5)	92 (42.7)	0.73	90 (54.6)	0.15
Coexisting conditions					
Diabetes mellitus	59 (48.8)	66 (34.2)	0.01	57 (34.6)	0.02
Hematologic disorder	1 (0.8)	1 (0.5)	1.00	2 (1.2)	1.00
HIV infection	0 (0.0)	1 (0.5)	1.00	0	1.00
Hypertension	64 (52.9)	75 (38.9)	0.02	80 (48.5)	0.48
Liver disease	4 (3.3)	1 (0.5)	0.07	2 (1.2)	0.25
Malignancy	15 (12.4)	14 (7.3)	0.16	13 (7.9)	0.23
Obesity	10 (8.3)	12 (6.2)	0.50	18 (10.9)	0.55
Peripheral vascular disease	12 (9.9)	3 (1.6)	0.002	9 (5.5)	0.17
Pulmonary disease	21 (17.4)	23 (11.9)	0.19	32 (19.4)	0.76
Renal disease	19 (15.7)	9 (4.7)	0.002	13 (7.9)	0.06
Transplant	1 (0.8)	0	0.39	0	0.42
Tobacco use	16 (13.2)	20 (10.4)	0.47	24 (14.6)	0.86
Alcohol abuse	4 (3.3)	2 (1.0)	0.21	6 (3.6)	1.00
Hospital-related risk factors					
Treatment at the academic tertiary care hospital	94 (77.8)	125 (64.8)	0.02	109 (66.1)	0.04
LOS before surgery, median, IQR	1, 0–4	0, 0–3	0.02	0, 0–2	0.01
LOS before culture, median, IQR	8, 5–14	NA	NA	5, 3–10	<0.001
Proportion of patients with an ICU stay before surgery	11 (9.1)	13 (7.9)	0.83	18 (9.3)	1.0
ASA score, median, IQR	3, 3–4	3, 2–4	0.03	3, 2–4	0.15
Duration of surgery (min), median, IQR	240, 166–305	194, 113–276	0.004	202, 116–285	0.01
Wound class, median, IQR	1, 1–1	1, 1–1	0.82	1, 1–1	0.36
NNIS Risk Index, median, IQR	1, 1–2	1, 1–1	0.002	1, 1–2	0.06

^a^LOS, length of stay; IQR, interquartile range; ASA, American Society of Anesthesiologists-Physical Status score; NNIS, National Nosocomial Infections Surveillance System.

**Table A2:** Study 1: Adjusted outcomes models for methicillin-resistant *Staphylococcus aureus* (MRSA) surgical site infection (SSI) compared to uninfected control patients^a^

Variable	Deaths	Length of stay^b^	Cost^c^
	OR (95% CI)	OR^d^ (95% CI)	OR (95% CI)
MRSA	11.4 (2.8 to 34.9)	3.2 (2.7 to 3.7)	2.2 (2.0 to 2.6)
ASA score^e,f^		1.3 (1.2 to 1.5)	ASA score = 4 3.7 (1.5 to 8.9)
			ASA score = 2 2.0 (1.4 to 2.9)
			ASA score = 3 3.0 (2.1 to 4.3)
			ASA Score = 4 4.1 (2.8 to 6.0)
>73 y of age	4.8 (2.0 to 11.6)		
Operative duration (min)^g^			
211–400		(0.9 to 1.3)	1.4 (1.2 to 1.7)
401–590		1.7 (1.2 to 2.4)	2.2 (1.6 to 3.1)
>590		1.8 (1.1 to 2.9)	2.6 (1.6 to 4.0)
Length of stay before surgery^h^			
7–13 d		1.6 (1.1 to 2.1)	1.7 (1.3 to 2.3)
14–20 d		3.6 (1.4 to 9.6)	5.6 (2.3 to 13.4)
>20 d		0.7 (0.2 to 2.6)	1.2 (0.3 to 4.3)
Intensive care unit stay before surgery			1.5 (1.2 to 2.0)
Tertiary care hospital			1.5 (1.2 to 1.7)

^a^OR, odds ratio; CI, confidence interval; ASA, American Society of Anesthesiologists -Physical Status. ^b^Model includes the following confounding variables: admission to the tertiary care hospital, diabetes, and renal disease. ^c^Model includes the following confounding variable: renal disease. ^d^For length of hospital stay and cost, OR represents multiplicative effect ^e^Length of stay increases by 1.3-fold for each point increase in ASA score. ^f^For cost, reference category is ASA score = 1. ^g^Reference category is operative duration < 211 min. ^h^Reference category is length of stay before surgery < 7 d.

**Table A3:** Study 1, adjusted outcomes models for methicillin-resistant *Staphylococcus. aureus* (MRSA) surgical site infections (SSI) compared to patients with methicillin-resistant *S. aureus* (MSSA) SSI^a^

	Deaths^b^	Length of Stay^c^	Cost^d^
Variable	OR (95% CI)	OR (95% CI)^e^	OR^e^ (95% CI)
MRSA	3.4 (1.5 to 7.7)	1.2 (1.0 to 1.5)	1.2 (1.0 to 1.4)
ASA score^f^	ASA score = 4 5.1 (2.1 to12.5)	ASA score = 2 0.9 (0.5 to 1.7)	ASA score = 2 1.0 (0.7 to 1.5)
		ASA score = 3 1.6 (0.9 to 2.9)	ASA score = 3 1.4 (1.0 to 2.1)
		Asa score = 4 1.8 (1.0 to 3.5)	ASA score = 4 2.1 (1.4 to 3.2)
Age > 61 years	3.0 (1.2 to 7.3)		
Operative duration, min^g^			
206–381		1.3 (1.0 to 1.6)	1.4 (1.1 to 1.6)
382–557		1.3 (0.8 to 2.1)	1.8 (1.3 to 2.5)
>557		1.1 (0.5 to 2.6)	1.6 (0.9 to 2.8)
Length (d) of stay before infection^h^			
11–20		1.4 (1.0 to 1.8)	1.6 (1.3 to 2.0)
21–30		1.6 (1.0 to 2.7)	1.7 (1.2 to 2.5)
>30		1.3 (0.5 to 3.1)	1.8 (0.9 to 3.8)
Renal disease		1.5 (1.0 to 2.2)	
Length (d) of intensive care unit stay before infection^i^			
8–14			1.8 (1.1, 2.8)
15–21			2.1 (1.1, 8.8)
>21			1.9 (0.4, 8.0)
Tertiary care hospital			1.3 (1.1, 1.6)

^a^OR, odds ratio; CI, confidence interval; ASA, American Society of Anesthesiologists -Physical Status. ^b^Model includes the following confounding variable: operative duration >222 min. ^c^Model includes the following confounding variables: admission to tertiary care hospital and diabetes. ^d^Model includes the following confounding variables: diabetes and renal disease. ^e^For length of hospital stay and cost, OR represents multiplicative effect. ^f^For deaths, reference category is ASA score < 1; for length of stay and cost, reference category is ASA score = 1. ^g^Reference category is operative duration < 206 min. ^h^Reference category is length of stay prior to infection < 11 d. ^i^Reference category is intensive care unit length of stay prior to infection < 8 d.

**Table A4:** Study 2, patient characteristics, vancomycin-resistant enterococci (VRE) wound infections, controls not infected with enterococci, and controls with vancomycin-susceptible enterococci (VSE) wound infections, bivariate analyses

Variable	Cases, VRE wound (%) (n = 99)	Controls, not infected (%) (n = 280)	P Value (VRE vs. controls not infected)	Controls, VSE (%) (n = 213)	p value (VRE vs. VSE)
Age, mean (y)	60.3	63.6	0.09	59.1	0.51
Sex (female)	46 (46)	124 (44.3)	0.7	127 (59.6)	0.03
Main diagnosis					
Orthopedic condition	11 (11)	30 (10.7)		18 (8.4)	
Cardiovascular condition	25 (25)	117 (41)		61 (28.6)	
Endocrine disorder	3 (3)	6 (2.1)		4 (1.9)	
Gastrointestinal disorder	25 (25)	60 (21.4)		62 (29.1)	
Genitourinary disorder	6 (6)	12 (4.2)		9 (4.3)	
Infectious disease	16 (16)	6 (2.1)		20 (9.4)	
Hematologic disease	0 (0)	2 (.7)		0	
Neurologic disease	11 (11)	32 (11.4)		34 (16)	
Pulmonary disease	2 (2)	14 (5)		5 (2.4)	
Coexisting conditions					
Cardiovascular disease	73 (74)	204 (72.9)	0.86	150 (70.4)	0.55
Lung disease	11 (11)	33 (11.7)	0.9	26 (12.2)	0.78
Diabetes mellitus	67 (67.7)	139 (49.6)	0.002	127 (59.6)	0.17
Organ transplant recipient	14 (14)	21 (7.5)	0.08	18 (8.4)	0.12
Renal disease	18 (18.2)	39 (14)	0.7	28 (13.2)	0.24
Malignancy	7 (7.1)	27 (9.6)	0.5	32 (15)	0.05
AIDS	2 (2)	2 (0.7)	0.27	0	0.1
Hepatobiliary disease	16 (16.6)	40 (14.3)	0.8	31 (14.5)	0.71
Charlson comorbidity score, mean	3.17	2.66	0.07		
Hospital-related risk factors					
Transfer from another institution	34 (34.3)	102 (36.4)	0.5	34 (16)	<0.001
Surgery	29 (29.3)	94 (33.6)	0.08	90 (42.3)	0.03
Admission to ICU	26 (26.2)	58 (20.7)	0.9	53 (33.3)	0.8

**Table A5:** Study 2, adjusted outcomes models for vancomycin-resistant enterococcus (VRE) wound infection compared to uninfected control patients^a^

Variable	Deaths^b^		Length of Stay^c^		Cost^d^
	OR (95% CI)	Variable	OR^e^ (95% CI)	Variable	OR^e^ (95% CI)
VRE infection	2.0 (0.8 to 5.2)	VRE infection	1.8 (1.3 to 2.4)	VRE infection	1.5 (1.3, 1.8)
		Transfer from another hospital	1.5 (1.2 to 1.9)	Surgery^e^	1.4 (1.1, 1.8)
		Renal disease	2.0 (1.5 to 2.7)		
		Malignancy	0.7 (0.5 to 0.9)		
		Intensive care unit stay^f^	2.3 (1.6 to 3.3)		

^a^OR, odds ratio; CI, confidence interval. ^b^Model includes the following confounding variables: intensive care unit (ICU) stay and number of coexisting conditions. ^c^Model includes the following confounding variable: propensity score (i.e., likelihood of being a VRE case). ^d^Model includes the following confounding variables: propensity score [i.e., likelihood of being a VRE case (Appendix)] and length of stay before infection (index date for controls). ^e^For length of hospital stay and cost, OR represents multiplicative effect. ^f^Before infection for cases and before index date for controls.

**Table A6:** Study 2, adjusted outcomes models for vancomycin-resistant enterococcus (VRE) wound infection compared to control patients with wound infection due to vancomycin-susceptible enterococcus (VSE)^a^

Variable	Deaths^b^		Length of Stay^c^		Cost^d^
	Odds Ratio (OR) (95% Confidence Interval [CI])	Variable	OR^e^ (95% CI)	Variable	OR^e^ (95% CI)
VRE	2.5 (1.1, 6.1)	VRE	1.1 (0.9, 1.4)	VRE	1.4 (1.2, 1.6)
Intensive care unit stay (ICU)^f^	9.0 (3.0, 27.4)	ICU stay^f^	1.8 (1.3, 2.5)	Surgery^f^	1.2 (1.1, 1.3)

^a^OR, odds ratio; CI, confidence interval; ICU, intensive care unit. ^b^Model includes the following confounding variables: gender and surgery before infection. ^c^Model includes the following confounding variable: malignancy and length of stay before infection. ^d^Model includes the following confounding variables: length of stay before cohort inclusion. ^e^For length of hospital stay and cost, OR represents multiplicative effect. ^f^Before infection for cases and before index date for controls.
